# Cationic Starch and Polyvinyl Alcohol Composite Sheets Produced by Extrusion-Calendering

**DOI:** 10.3390/foods15193360

**Published:** 2026-09-22

**Authors:** Juliano Zanela, Ana Paula Bilck, Gracielle Johann, Marianne Ayumi Shirai, Maria Victoria Eiras Grossmann, Fabio Yamashita

**Affiliations:** 1 Department of Food Science and Technology, Universidade Estadual de Londrina, Rod. Celso Garcia Cid (PR 445), km 380, P.O. Box 6001, Londrina 86051-990, PR, Brazil; apbilck@gmail.com (A.P.B.); victoria@uel.br (M.V.E.G.); fabioy@uel.br (F.Y.); 2 Bioprocess Engineering and Biotechnology Course, Federal University of Technology—Paraná, Estrada para Boa Esperança, Km 04, Dois Vizinhos 85660-000, PR, Brazil; grajohann@gmail.com; 3 Department of Food Technology, Federal University of Technology—Paraná, João Miguel Caram, 731, Londrina 86036-700, PR, Brazil; marianneshirai@utfpr.edu.br

**Keywords:** biodegradable polymers, calendering, mechanical properties, modified starch, PVA

## Abstract

This study evaluated the effect of cationic cassava starch with different degrees of substitution (DS: 0.019, 0.027, and 0.033 mol/mol) and polyvinyl alcohol (PVA) with different hydrolysis degrees (HD: 88 and 98%) on the properties of sheets produced by extrusion-calendering, using glycerol as a plasticizer. Machine learning (ML) models were used to predict sheet properties. The HD influenced material properties more significantly than the DS of cationic starch, with higher HD PVA yielding superior mechanical performance regardless of starch type. The sheets exhibited high thermal stability and processing continuity during extrusion-calendering, demonstrating laboratory-scale production viability. ML demonstrated that Gradient Boosting models better predicted tensile strength and elongation at break with R^2^ ~0.98 and MAPE of the 7.1 and 2.8%, respectively; the Young’s modulus was adequately predicted by the Decision Tree model (R^2^ ~0.96 and MAPE ~8.1%) and the apparent opacity was better predicted by the Extreme Gradient Boosting model (R^2^ ~0.90 and MAPE ~4.7%). For these adjustments, the HD was more important than DS when predicting the properties of the sheets, as observed experimentally.

## 1. Introduction

Synthetic plastics are widely used in our daily lives, but they cause serious environmental problems when disposed of. In recent years, research on biodegradable materials has increased as an alternative to petroleum-based plastics [1,2].

Nowadays, several polymers can be used to replace conventional plastics, like polyvinyl alcohol—PVA, a polymer with high technological potential because it is a melt-processable polymer that possesses excellent optical and physical properties. It is used in a wider range of industrial fields; however, PVA is relatively expensive compared to conventional non-biodegradable polymers [3]. Therefore, blending PVA with starch can reduce costs and compete with conventional polymers, as shown in several works. Considering the use of modified starch, cationic starch, due to its positive functional groups, can facilitate interactions with PVA chains, improving mechanical properties by increasing tensile strength while decreasing water vapor permeability [4,5].

Starch has some limitations in its native form, so to improve specific properties, there are physical, chemical, or enzymatic methods to produce modified starches. Cationic starches are widely used in numerous industrial applications in the food, paper, and textile industries, and they are produced through the etherification reaction of starch with compounds containing tertiary or quaternary ammonium salts [6,7].

Usually, the cationic starches present degrees of substitution (DS) lower than 0.2, and the production process can be made by the dry or wet route; the physical and chemical properties and the morphology of starch are changed after the cationization, mainly at higher DS values [8,9]. Chivrac and collaborators produced cationic starch-based nano-biocomposites using sepiolite [10] and montmorillonite [11]. According to the authors, the cationic starch favored the exfoliation of sepiolite and montmorillonite nanoclays due to its adequate polarity, which enhanced exfoliation, demonstrating the potential use of cationic starch in the production of biodegradable materials.

Starches from several botanical sources like pea [12], corn [13,14], potato [15], and cassava [16,17,18,19] were used in blends with PVA, as well as modified starches [20] to produce biodegradable materials. Zanela et al. (2020) [21] tested five cassava starches (one native as a control and four modified: distarch phosphate, acetylated distarch phosphate, acetylated distarch adipate, and oxidized starch) and two PVA grades (fully and partially hydrolyzed) in the extruded-calendering sheet production. In general, the use of modified starches did not alter the functional properties of the sheets, and the PVA hydrolysis degree was the main factor responsible for the improvement in the mechanical properties. These findings motivate research into other modified starches, aiming to obtain biodegradable materials with enhanced properties.

Polyvinyl alcohol is produced through the hydrolysis of poly(vinyl acetate) in a methanolic solution, and PVA with different hydrolysis degrees can be produced by replacing the acetate groups (-OCOCH_3_) with hydroxyl groups (-OH). PVA is a non-ionic polymer, but it can gain a negative charge due to the formation of a resonant structure [22,23]. Thus, we hypothesize that the negative charge in the PVA structure could interact with cationic starch, improving the blend’s characteristics compared to the native starch or fully hydrolyzed PVA. The introduction of cationic quaternary ammonium groups at low DS disrupts native starch crystallinity and promotes electrostatic interaction with the PVA matrix without compromising polymer biodegradability, and improves phase compatibility and mechanical properties in PVA and starch blends.

Machine learning (ML) is a branch of artificial intelligence that has been applied to create models (knowledge) that can effectively learn from existing data and discover non-linear correlations in experimental data. Compared with conventional statistical modeling techniques, such as linear regression and response surface methodology, artificial intelligence-based methods have shown superiority as modeling techniques for datasets showing non-linear relationships. Particularly in materials science, ML has demonstrated substantial efficacy in predicting property profiles and optimizing formulations for polymer composites [24,25,26].

To the best of our knowledge, there are no contributions on the production of sheets by extrusion-calendering using cationic starch with different DS and PVA, and the use of ML to predict the sheets’ properties. Therefore, this work aimed to study the effect of cationic cassava starch with different degrees of substitution and PVA with different hydrolysis degrees on the properties of composite sheets of starch/PVA produced by extrusion-calendering.

## 2. Materials and Methods

### 2.1. Materials

Four cassava starches used were supplied by Agricola Horizonte Ltda (Novo Três Passos, Brazil): one native starch—NS—and three cationic cassava starch with different DS, Superion 150, coded as CS19 (DS = 0.019 mol/mol), Superion 200, coded as CS27 (DS = 0.027 mol/mol) and Superions 300, coded as CS33 (DS = 0.033 mol/mol). The degrees of substitution were obtained for the analysis data sheet for each sample provided by the producer industry. There were used PVAs supplied by Sekisui Chemical (Tokyo, Japan), with different hydrolysis degrees—HD—and molecular weights—MW—(based on their viscosity in 4% aqueous solution): Selvol™ 540—S540 (HD: 88%, viscosity: 49 cP) and Selvol™ 325—S325 (HD: 98%, viscosity: 31 cP). The glycerol was supplied by Dinamica (São Paulo, Brazil).

### 2.2. Sheet Production

All eight formulations were composed of 40 wt% native or cationic starch, 25 wt% PVA, and 35 wt% glycerol. The concentration of the components was determined by preliminary tests. After manual homogenization, the blends were placed in a vacuum oven (model Q819V2, Quimis, Brazil) with a vacuum pressure of 0.085 MPa for 90 min at 85 °C to incorporate the glycerol using a method adapted from Jang & Lee (2003) [27]. After this step, the blends were extruded in a single-screw extruder (model EL-25, BGM, São Paulo, Brazil) with a screw diameter of 25 mm and an L/D ratio of 28. The screw speed used was set at 30 RPM, the temperature profile used was set at 90/180/200/190 °C from feeder to die zone, and a six-hole cylindrical matrix (2 mm) was used for pellet production.

After pelletization, the blends were extruded in a co-rotating twin-screw extruder (model D-20, BGM, Brazil) with a screw diameter of 20 mm and L/D ratio of 35, screw speed of 110 RPM, and temperature profile of 90/160/190/190/190 °C from feeder to flat die. The samples were extruded using a flat die with 0.8 mm aperture and 320 mm length, coupled with a 3-roll water-cooled calender (AX Plásticos, Diadema, Brazil) for sheet production.

The blend formulations were identified by the acronym of the starch type followed by the PVA type, e.g., CS19-S540: blend using cationic cassava starch with DS of 0.019 mol/mol (CS19) and PVA Selvol™ 540.

### 2.3. Mechanical Properties

The tensile strength, Young’s modulus, and elongation at break were analyzed according to the ASTM D882-02 [28] method, with some modifications, using a universal testing machine with a load cell of 5 kN (model DL2000, EMIC, São José dos Pinhais, Brazil) with an initial distance between the grips of 40 mm, and a crosshead speed of 0.8 mm.s^−1^. Ten specimens from each formulation were cut (80 mm in length and 25 mm in width) and conditioned at 53 ± 2%RH and 23 ± 2 °C for 72 h before analysis.

### 2.4. Water Vapor Permeability (WVP)

Water vapor permeability (WVP) analysis was determined according to the ASTM E96-009 standard (gravimetrically) [29]. The measurements were performed in triplicate, using a relative humidity gradient of 33–64% RH.

### 2.5. Weight Loss in Water (WLW)

The materials’ weight loss in water (WLW) was performed as described by Olivato et al. (2012) [30]. The WLW was performed in triplicate and expressed as the percentage of the original mass (M_i_) and the film’s final mass (M_f_) after immersion in water for 48 h at 25 °C (Equation (1)).

(1) $$WLW=\frac{M_{i}-M_{f}}{M_{i}}\times 100$$

### 2.6. Apparent Opacity and Color Difference

The apparent opacity (Op) and color difference (∆E*) were determined according to the method described by Maria et al. (2008) [31]. The Op values were measured in triplicate using a colorimeter (BYK Gardner, Color-Guide, Geretsried, Germany) with the illuminant D65 (daylight) and a visual angle 10°. The Op was calculated as the ratio of the sample opacity over black (Op_b_) and white standards (Op_w_) (Equation (2)) and represented on an arbitrary scale (0–100%).

(2) $$Op=\frac{{Op}_{b}}{{Op}_{w}}\times 100$$

The ∆E* was calculated according to Equation (3), where ∆L*, ∆a*, and ∆b* are the differences between the samples measured under the white standard and the white standard alone.

(3) $$∆E*=\sqrt{{\left({∆L}^{*}\right)}^{2}+{\left({∆a}^{*}\right)}^{2}+{\left({∆b}^{*}\right)}^{2}}$$

### 2.7. X-Ray Diffraction (XRD)

The X-ray diffraction (XRD) analysis was performed using a diffractometer (Panalytical, X’Pert PRO MPD, Almelo, The Netherlands) that emitted copper Kα radiation (λ = 1.5418 Å). The anode radiation was generated at 40 kV and 50 mA and was monochromatized using a 20 mA current. The baseline (amorphous region) was manually defined in order to reproduce a baseline following a parabola shape connecting the lowest points of the diffractogram. The relative crystallinity of each film was calculated by dividing the peak area by the total area under the curve for the crystalline region and the amorphous region (the total area under the curve minus the baseline), as described by Müller et al. [32]. The relative crystallinity index was calculated using Origin software (OriginLab Corporation, 8.5.1 Version, Northampton, MA, USA).

### 2.8. Scanning Electron Microscopy (SEM)

The micrographs of the biodegradable sheets were recorded using a scanning electron microscope (SEM) (FEI, Quanta 200, Columbia, MD, USA). The sheets were fractured in liquid nitrogen, attached to aluminum supports, and coated with gold (BAL-TEC SCD 050 sputter coater, Leica Microsystems, Wetzlar, Germany) (40–50 nm in thickness) at 25 °C and a pressure of 2.105 Torr for 180 s. The fracture surfaces of the sheets were analyzed using an accelerating voltage of 30 kV, working distance of about 10 mm, and aperture size of 30 μm [21].

### 2.9. Fourier Transform Infrared Spectroscopy (FT-IR)

The sheets were dried over anhydrous calcium chloride salt for one week and analyzed in a Fourier transform infrared spectrophotometer (FT-IR) (IRPrestige 21, Shimadzu, Kyoto, Japan) using a horizontal attenuated total reflection (ATR) module operating over the spectral range of 4000 cm^−1^–750 cm^−1^. The Standard Normal Variate Transformation—SNV—of the spectra was used to reduce particle size, light scattering, and multicollinearity, improving the spectra interpretation, according to Barnes et al. (1989) [33].

### 2.10. Thermogravimetric Analysis (TGA)

After drying the material at 0% RH in anhydrous calcium chloride, the samples were scanned using a TGA-50 (Shimadzu, Kyoto, Japan) from 30 to 600 °C, with a heating rate of 10 °C·min^−1^ under a nitrogen atmosphere (20 mL·min^−1^). Both thermogravimetric analysis—TG—and derivative thermogravimetric analysis—DTG—were performed [16].

### 2.11. Machine Learning in Predicting Film Properties

In order to predict film properties, the variables degree of substitution, DS, and degree of hydrolysis, HD, were used as input variables in machine learning models, whose outputs were tensile strength, Young’s modulus, elongation, water vapor permeability, mass loss in water, apparent opacity, and color difference. The entire infrastructure was implemented in Python 3.0, using the Scikit-learn library [34]. The dataset, consisting of 80 observations (10 experimental replicate measurements collected across 8 formulations with different combinations of DS and HD), was divided into two sets, one for training and the other for testing, in a 70:30 ratio, followed by data normalization. The regression methods Multi-Layer Perceptron Neural Network (MLP), Decision Tree (DT), Random Forest (RF), Gradient Boosting (GB), Extreme Gradient Boosting (XGB), and K-Nearest Neighbor (KNN) were tested using two-fold cross-validation. The model hyperparameters (Table 1) were optimized using the GridSearch and RandomizedSearch tools.

The performance of the models was evaluated by the statistical metrics coefficient of determination (R^2^), mean squared error (MSE), mean absolute percentage error (MAPE), mean absolute error (MAE), and root mean squared error (RMSE). In turn, the importance of the input variables in the predictive capacity of film properties was evaluated using the Random Forest model.

### 2.12. Statistical Analysis

Data were analyzed using STATISTICA 7.0 (StatSoft Inc., Tulsa, OK, USA). A two-way Analysis of Variance (Two-Way ANOVA) was performed to evaluate the main effects of starch type (four levels), PVA grade (two levels), and their interaction terms (starch x PVA) on the sheet’s properties. Mean comparisons were conducted using Tukey’s test at a 5% significance level (*p* < 0.05). All reported experimental replicates correspond to multiple specimens systematically sampled along the continuous extrusion-calendered sheet obtained from each formulation.

## 3. Results and Discussion

The sheets produced by extrusion-calendering had good processability and handleability, and were visually homogeneous and continuous, with material thicknesses as shown in Table 2. Variability in thickness is evident. This is likely due to rheological differences among the formulations, necessitating adjustments to calendering production settings and resulting in variations in material thickness. No clear pattern is observed in the results regarding the types of starch and PVA tested.

### 3.1. Mechanical Properties

The sheets produced with PVA with the higher hydrolysis degree (S325-HD = 98%) had the highest values for mechanical properties (tensile strength, elongation at break and Young’s modulus) when compared to the sheets produced with PVA with the lower HD (S540-HD = 88%) for all starches tested, while cationic starches were similar to or lower than the native one (Figure 1 and Supplementary Material).

Limpan et al. (2012) [35] reported the same behavior for casting films of fish myofibrillar proteins/PVA blends, in which films with higher HD PVA had better mechanical properties than films produced with lower HD PVA. According to the authors, higher HD PVA has more hydroxyl groups in its structure, which favor hydrogen bonding between starch and PVA molecules, leading to stronger interactions and, consequently, more rigid structures. Liu et al. (2023) [36] also observed the same tendency in the extruded starch and PVA foams when PVA with a higher hydrolysis degree was used.

Similar results were obtained in sheets of cassava starch blended with PVA with five different HDs [37]. According to the authors, the PVA’s hydrolysis degree was responsible for increasing the materials’ rigidity. The molecular weight was related to tensile strength and elongation at the break of the materials, with similar results in both cited and present works.

The hypothesis of the higher interaction between the acetate groups of partially hydrolyzed PVA and the cationic starch was not observed because the mechanical properties of the materials produced with cationic starches were equal to or lower than those with native starch. Moreover, the residual acetate groups in the PVA S540 can potentially weaken hydrogen bonding, thereby overlapping any electrostatic interaction between the starch’s cationic groups and the PVA’s partially hydrolyzed acetate groups.

According to Briscoe et al. (1999) [38], the residual acetate groups of the partially hydrolyzed PVA have a hydrophobic nature, which weakens the hydrogen bonds intra- and intermolecular of the polymers, reducing the mechanical properties of the sheets. This weakening of hydrogen bonds was probably more deleterious for the mechanical performance of the blend than a possible interaction of cationic starch and the acetate groups in the PVA molecule.

Regardless of the PVA used, the cationic starch shows a reduction in tensile strength compared with the native one, similar to what is observed by Sarak et al. (2022) [39], in films of pure native or cationic (DS = 0.02–0.033) cassava starches.

Wang et al. (2017) [40] produced blow films by extrusion with cationic starch and fully hydrolyzed PVA, with starch/PVA ratios ranging from 100:0 to 80:20 wt%. According to the authors, increasing the PVA content increased tensile strength, with values ranging from 2 to 3 MPa, lower than those obtained in the present work for the high HD PVA (S325). The same behavior was observed for elongation at break, with values ranging from 50 to 75%, lower than those observed in the present work for both types of starch, probably because of the lower PVA content. The authors did not provide information on the concentration of the plasticizer. Sarak et al. (2022) [39] observed that native corn starch presented higher values than cationic ones (DS = 0.02–0.033); the same was observed in the present work, in that the increase in DS value leads to the reduction in tensile strength regardless of the PVA grade.

### 3.2. Water Vapor Permeability (WVP)

The WVP of the biodegradable sheets ranged from 2.7 to 3.8 × 10−10 g.m^−1^.s^−1^.kPa^−1^ (Table 2), and these values were similar to those reported by Zanela et al. (2020) [16], who used the same PVA grades with five different starches (one native and four modified), with WVP ranging from 2.8 to 3.9 × 10^−10^ g.m^−1^.s^−1^.kPa^−1^. Wang et al. (2017) [40], in blown films of cationic starch and PVA, reported higher WVP, ranging from 4.5 to 7.0 × 10^−10^ g.m^−1^.s^−1^.kPa^−1^, for PVA content that ranged from 0 to 20%. According to the author, these variations were due to structural modifications of the polymeric network and interaction between the hydroxyl groups of both polymers because the water vapor permeation depends on both the diffusivity and solubility of water in the polymeric matrix. Neither starch cationization nor PVA grade affected the sheets’ WVP, likely because all blend components (starch, PVA, and glycerol) are hydrophilic.

### 3.3. Weight Loss in Water (WLW)

The PVA grade was the main promoter of the solubility, similar to what was observed by Petry et al. (2024) [41], in films with different proportions of cationic starch:PVA. This effect is more pronounced when starch with a high DS is used. The biodegradable sheets produced with PVA S540 (HD = 88%) had lower weight loss in water (WLW) than those with PVA S325 (HD = 98%) (Table 2), ranging from 34 to 52%, characterizing these materials as soluble in water because, as previously discussed, the blend components (starch, PVA, and glycerol) are hydrophilic, and the insoluble fraction is formed by the entangled high-molecular-weight polymer matrix and crystalline PVA domains that resist room-temperature dissolution. It is important to emphasize that glycerol is a highly hydrophilic, low-molecular-weight polyol that readily diffuses into aqueous media, acting as the primary contributor to early-stage mass loss during solubility testing. Furthermore, by disrupting inter- and intramolecular hydrogen bonds between starch and PVA chains to increase matrix free volume, glycerol facilitates water ingress, which in turn accelerates the leaching of soluble polymer fractions.

The values of WLW obtained in this study are lower than those reported by Zanela et al. (2015) [17], which obtained values ranging from 54 to 75% for native starch/PVA, but they used a PVA with a lower molecular weight than those used in this work. Similar results were reported by Zanela et al. (2020) [21], testing the same PVA’s grades in modified cassava starch sheets, with WLW ranging from 36 to 52%. The lower WLW (34%) was similar to those reported by Soares et al. (2013) [42], for sheets produced with native cassava starch, poly (lactic acid), and glycerol (0.3 g of glycerol/g of starch) (starch:PLA 70:30 wt%), that obtained WLW of 35.2% despite the PLA being less hydrophilic than PVA.

While the WLW evaluated in this study reflects the fraction of soluble or eroded material after immersion, it is worth noting that water transport and structural swelling kinetics (water uptake) play a key role in biopolymer network stability. The degree of starch cationization and the PVA grade strongly modulate hydrogen bonding and crosslinking, directly influencing water penetration rates. In related starch-based networks, time-dependent water absorption kinetics are frequently described using empirical models, which provide insights into relaxation-driven versus diffusion-driven water transport [43,44]. Although swelling kinetic profiles were not experimentally measured in the present work, the structural integrity observed during immersion suggests that the cationized starch/PVA matrix maintained its cohesive sheet structure despite partial dissolution.

### 3.4. Apparent Opacity (Op) and Color Difference (∆E*)

The sheets’ apparent opacity (Op) ranged from 22 to 38% (Table 2), and the formulation CS19-S325 had the higher Op and NS-S540, CS27-S540, CS27-S325, and CS33-S325 than the lower Op, and consequently, they were the more translucent sheets. For ∆E* analysis (Table 2), the sheets NS-S325 and CS19-S325 presented higher ∆E* values, indicating that these sheets had the highest color difference when compared to the white standard. These films were yellowish; on the other hand, the sheets CS27-S540 and CS33-S325 had a lower ∆E* because of their higher transparency (low Op). The PVA grade and type of starch influenced the biodegradable sheets’ opacity and color, and the values were similar to those reported for biodegradable sheets of starch and PVA produced by extrusion, with Op ranging from 20 to 44% and ∆E* from 12 to 34 [21,37].

### 3.5. X-Ray Diffraction (XRD)

The X-ray diffractograms of the sheets were overall similar, with the crystallinity index (Table 2) showing little variation, ranging from 21.9 to 26.5%. Overall, sheets produced with PVA S325 (HD = 98%) exhibited slightly higher crystallinity than those produced with PVA S540 (HD = 88%). According to Tang & Alavi (2011) [3], in fully hydrolyzed PVA, the predominant presence of small hydroxyl groups allows for tight intermolecular hydrogen bond and efficient chain packing. Conversely, in partially hydrolyzed PVA, bulky residual acetate groups introduce steric impediment that disrupts chain alignment, thereby lowering crystallinity index.

The higher crystallinity ordering in PVA S325 blends aligns with the higher Young’s modulus observed in the mechanical properties section (Figure 1B). The lower volume of hydroxyl groups compared with acetate groups, together with differences in PVA viscosity and chain entanglement, facilitates the ordering of polymeric chains in ordered zones, resulting in a stiffer and less deformable material.

The sheets produced with PVA S325 show a peak near 20° and two peaks partially overlapped at 23° and 17.9°, while PVA S540 sheets have a broad peak near 20°, and the 23° and 17.9° peaks are likely overlapped by the peak at 20°. The absence or overlapping of these peaks supports the absence of phase separation in SEM images (Figure 3), as will be discussed later in Section 3.5. Zanela et al. (2015) [17] obtained similar results for native cassava starch/PVA extruded sheets, as well as Das et al. (2010) [45] and Boonsuk et al. 2020 [46], in oxidized cassava starch:PVA casting films.

Wang et al. (2017) [40] obtained similar diffractograms for films of PVA: cationic starch produced by blown extrusion. The diffraction pattern of native and cationic starches (Figure 2) was similar, and according to Qiu, Hu, and Peng (2013) [47], it indicates that the modification occurred mainly in the amorphous regions of the starch granules, and cationization of the starch did not influence the re-crystallization of the polymers after the extrusion process. Similar diffraction patterns were observed in cationic corn starch:PVA casting films obtained by Petry et al. (2024) [41], using 15 wt% of sorbitol as a plasticizer, with the presence of a broad peak near 20°, indicating a semicrystalline structure for the obtained material. The crystalline structure is critical for the mechanical properties of the sheets, and the higher crystallinity observed in S325 films can be directly related to the superior mechanical properties, regardless of the starch type used.

### 3.6. Scanning Electron Microscopy (SEM)

According to the SEM analysis (Figure 3), the sheet fracture is smooth, without cracks or pores, indicating good polymer compatibility because both PVA and starch are hydrophilic and have many hydroxyl groups, which favor interaction via hydrogen bonding. Top-surface SEM micrographs also revealed smooth, continuous, and homogeneous surfaces across all formulations, confirming that the calendering process imparted uniform surface quality without macro-defects or phase separation. This is in accordance with Zanela et al. (2015) [17,48] for cassava starch:PVA extruded sheets, Zanela et al. (2020) [21] for modified cassava starch:PVA extruded sheets, and with Luo et al. (2012) [49], for corn starch:PVA plasticized with glycerol.

On the other hand, Wang et al. (2017) [40] observed granules of starch partially melted in cationic starch/PVA blown films. However, the authors reported continuous film production, indicating that good compatibility between the polymers was also achieved in the present work.

**Figure 3:**
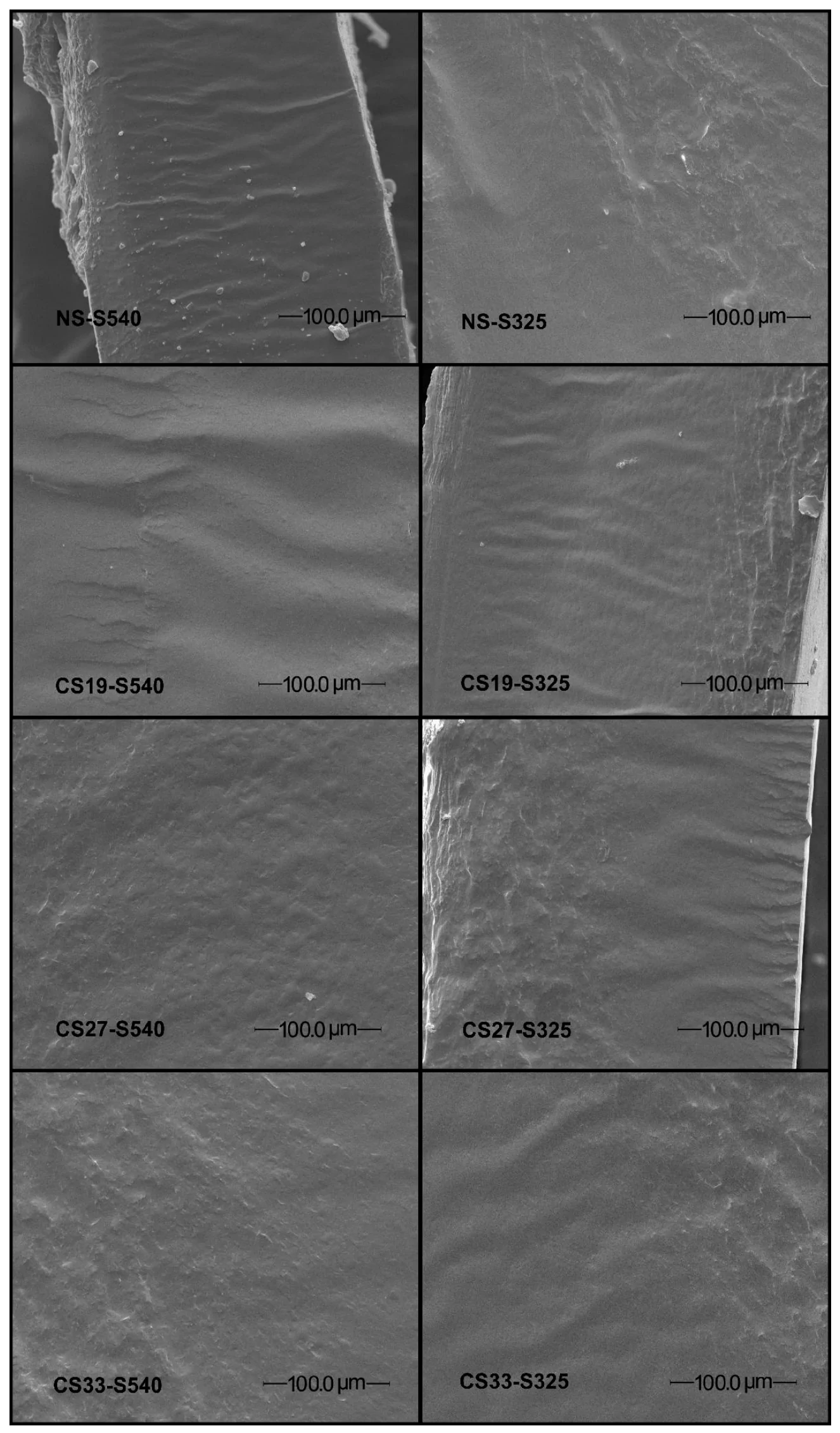
Scanning electron microscopy—SEM—micrographs of the fracture surfaces of the biodegradable sheets (magnification at 800×).

### 3.7. Fourier Transform Infrared Spectroscopy (FT-IR)

The spectra obtained in this study (Figure 4) are consistent with those described for starch/PVA blends produced by extrusion-calendering, in which bands characteristic of hydroxyl groups, carbonyl groups, and glycosidic bonds predominate, without the appearance of new functional groups indicative of extensive chemical modification of the matrix. The large absorption band near 3300 cm^−1^ was attributed to the stretching of hydroxyl groups present in the three components of the sheets (starch, PVA, and glycerol) [47,48].

All the sheets produced with PVA S540 presented absorption bands at 1730 cm^−1^ and 1245 cm^−1^, attributed to the stretching of C=O and C–O bonds, respectively, due to the residual acetate groups present in partially hydrolyzed PVA molecule [17,21,50]. It demonstrates that the acetate residual groups can weaken intra- and intermolecular hydrogen bonding, thereby restricting interactions between these groups and the cationic starch. Consequently, these findings suggest that the weakened molecular interactions directly impaired the mechanical performance. Specifically, compared to native starch, the use of cationic starches with higher degrees of substitution (CS27 and CS33) led to a noticeable reduction in both tensile strength and elongation at break.

Sin et al. (2010) [51] studied the hydrogen bond interactions in native cassava starch: fully hydrolyzed PVA, experimentally and through computational modeling, and the authors observed that starch:PVA binding is favorable and stable, indicating good compatibility between them. This corroborates the good compatibility observed in the SEM analysis, with no phase separations, pores, or cracks in the materials.

Although the SEM micrographs indicate good morphological homogeneity at the microscale, this observation alone does not fully prove polymer compatibility between starch and PVA matrix at the molecular level. To comprehensively evaluate interfacial compatibility, these morphological observations must be interpreted alongside structural, mechanical properties and crystallinity index.

### 3.8. Thermogravimetric Analysis (TGA)

According to Figure 5 (TG and DTG analysis), a similar weight-loss pattern is observed for both starch- and PVA-based biodegradable sheets, with three distinguishable weight-loss regions.

The first weight-loss region occurs at approximately 244–250 °C for S325 and S540 PVA, respectively. The main degradation stage occurs between 312 and 334 °C for PVA S325, and 316–325 °C for PVA S540, and the native starch shifts the main degradation stage to higher temperatures for both PVAs compared with the cationic starch. The third degradation step occurs at nearly 440 °C for all samples. The results are similar to those observed in other studies [12,17,37], among others.

The first degradation stage involves the evaporation or volatilization of residual bonded water and small-molecule volatiles such as glycerol; the second stage involves the degradation of both starch and PVA molecules; and the third stage involves the carbonization of organic matter.

The estimation of glycerol content in the sheets was calculated from TGA data. While TGA presents some challenges for absolute quantification due to the partial thermal overlap between glycerol volatilization and the early stages of polymer degradation, it can indeed provide a reliable estimate of the residual plasticizer. Based on the mass loss observed in the expected glycerol volatilization stage (typically between 150 °C and 280 °C), the estimated glycerol loss content in the extruded sheets is 5.52 wt% of glycerol due to the partial volatilization of the plasticizer during the thermo-mechanical extrusion process. Correcting the mass balance, the extruded sheets contain 42.34, 26.46, and 31.2 wt% starch, PVA, and glycerol, respectively.

### 3.9. Machine Learning in Predicting Film Properties

After testing all models, MLP, DT, RF, GB, XGB, and KNN, as well as optimizing their respective hyperparameters, the results presented in Table 3, Table 4, Table 5, Table 6, Table 7, Table 8 and Table 9 were obtained. In turn, Table 10 presents the results of the statistical metrics obtained for each film property.

From the results in Table 3, Table 4, Table 5, Table 6, Table 7, Table 8, Table 9 and Table 10, it can be observed that the GB model adequately predicted tensile strength and elongation at break properties, with R^2^ ~0.98 and MAPE of the 7.1 and 2.8%, respectively, in addition to reduced values for MSE, MAE, and RMSE. In turn, Young’s modulus was adequately predicted by the DT model DT (R^2^ ~0.96 and MAPE ~8.1%) and the apparent opacity was better predicted by the XGB model (R^2^ ~0.90 and MAPE ~4.7%). For the other properties, water vapor permeability, mass loss in water, and color difference, the tested models did not show an adequate fit.

The result of the analysis of feature importance in properties prediction, HD and DS, in the properties of the films is presented in Table 11.

From Table 11, it can be observed that the hydrolysis degree, HD, has the greatest importance across all evaluated properties, with values ranging from 0.53 (WVP and Op) to 0.87 (tensile strength). This result indicates that HD is the variable that contributes most to the model’s predictive capacity, and, consequently, to the output variables (film properties). This result corroborates the results obtained for the statistical analysis of film properties.

It is important to emphasize that while the HD of PVA significantly influences the inter- and intramolecular hydrogen bonding capacity, the two PVA grades used also differ in viscosity, which reflects variations in their molecular weight. Therefore, the changes observed in the physical, mechanical, and thermal properties of the sheets consider the combined effect of PVA viscosity and HD.

## 4. Conclusions

The hydrolysis degree—HD—and viscosity/molecular weight of polyvinyl alcohol is more relevant for the materials’ properties than the degree of substitution of cationic cassava starch, and the sheets produced with higher-HD PVA presented better mechanical properties and higher opacity and ΔE than those produced with lower-HD PVA, independently of the type of starch. Based on the machine learning model’s tensile strength, elongation at break, and Young’s modulus, apparent opacity of the sheets was successfully predicted, and results indicate that HD is the variable that contributes most to the model’s predictive capacity. The formulation of cationic starch/PVA blends demonstrated favorable processability via extrusion-calendering at the laboratory scale, yielding continuous and cohesive sheets without significant polymer degradation or loss of mechanical properties. However, further studies focusing on process scale-up, long-term production stability, and techno-economic evaluation are required to determine their industrial and commercial feasibility.

## Figures and Tables

**Figure 1:**
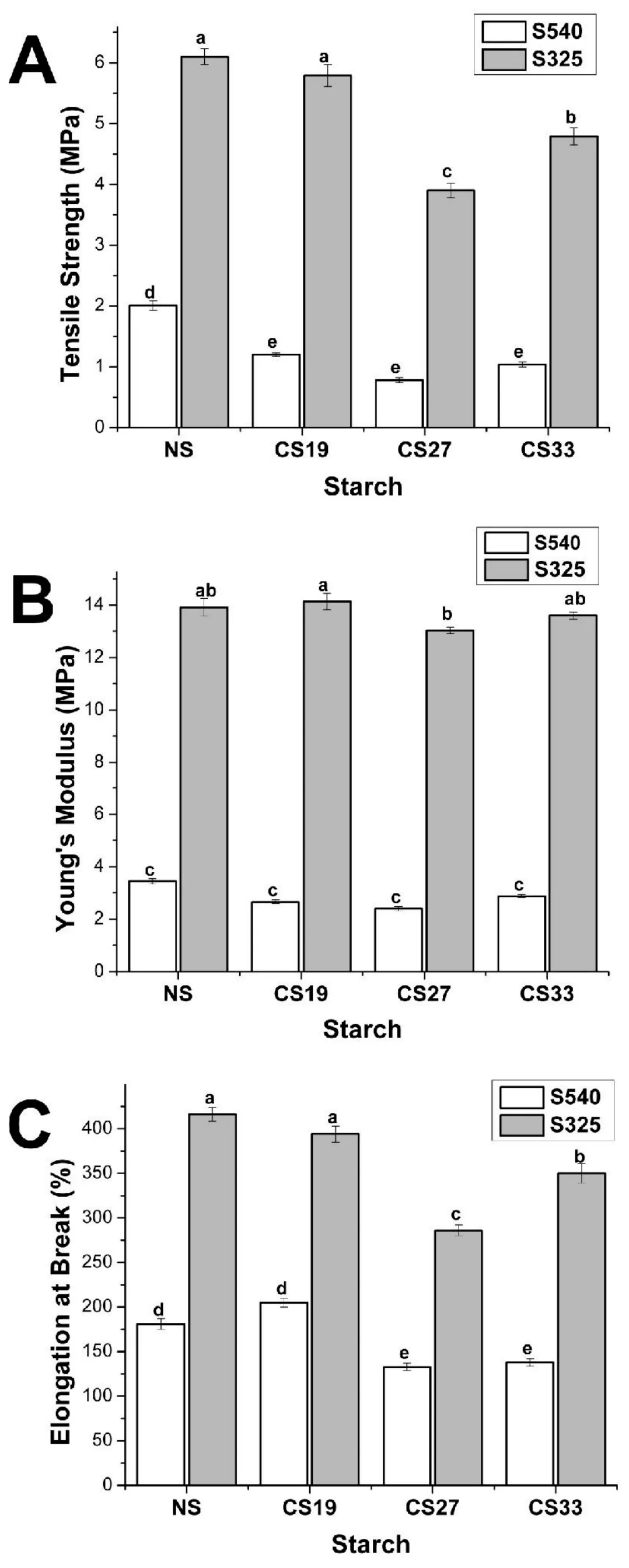
Mechanical properties of the biodegradable sheets: tensile strength (**A**), Young’s modulus (**B**), and elongation at break (**C**). Different letters represent significant difference (*p* ≤ 0.05) between the means obtained by Tukey’s test.

**Figure 2:**
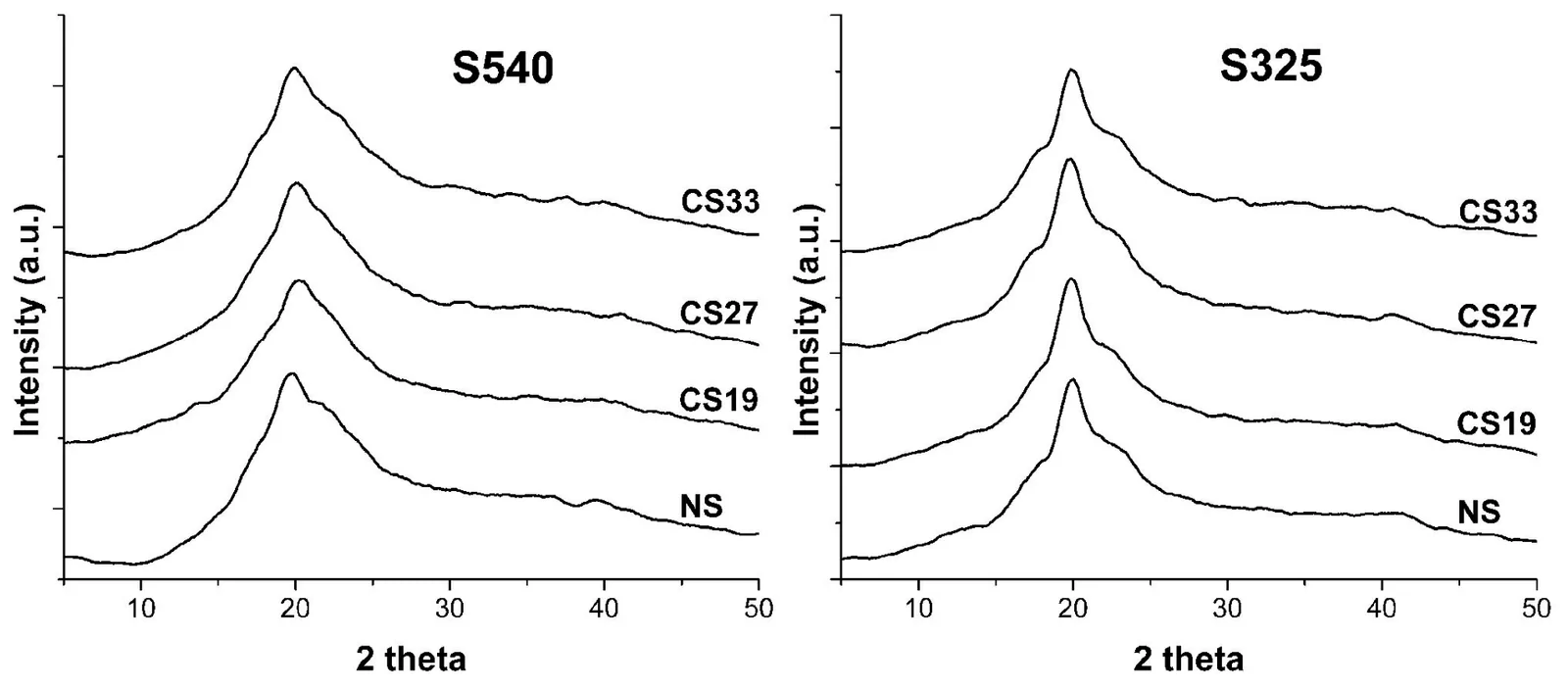
Diffractograms of the biodegradable sheets.

**Figure 4:**
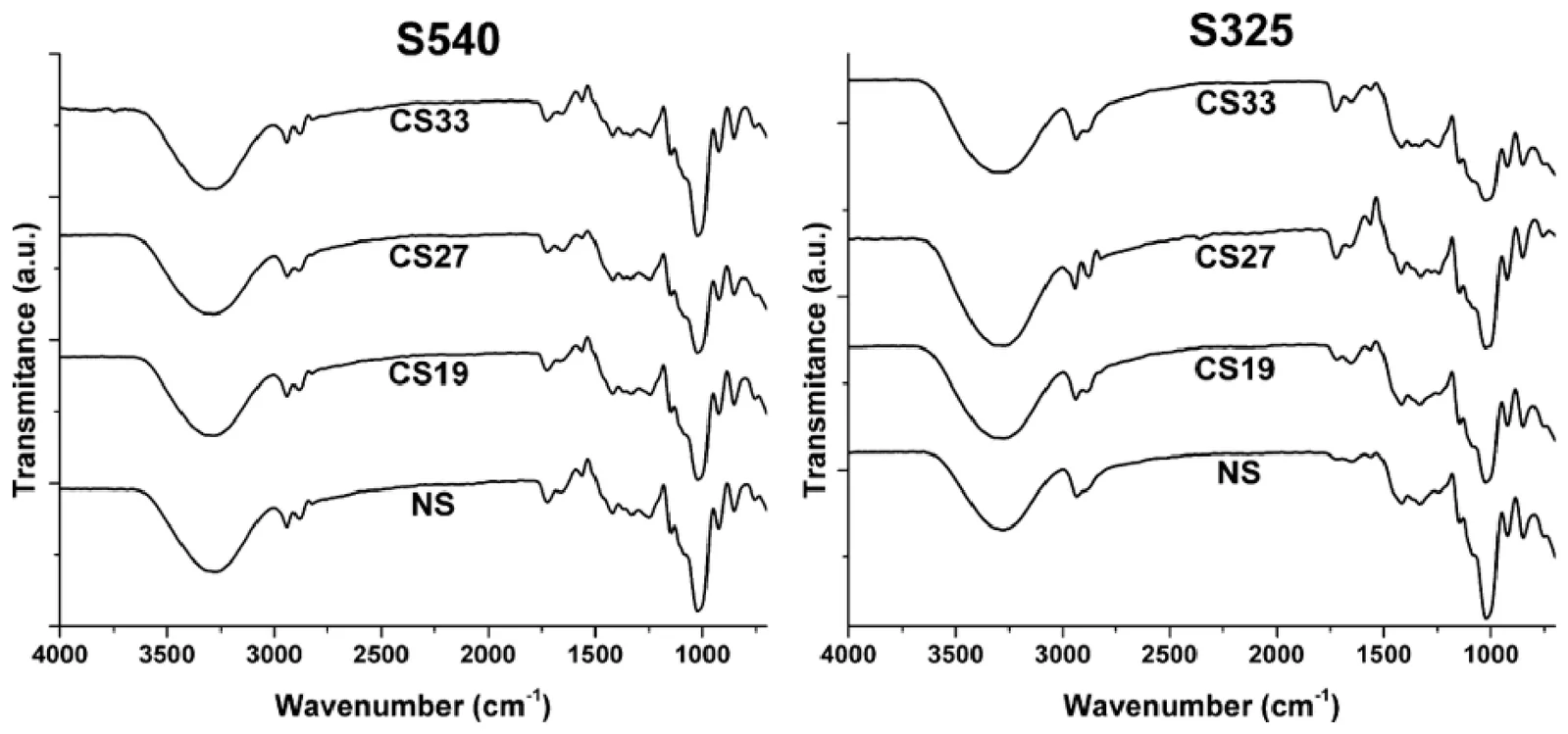
Fourier Transform Infrared Spectroscopy—FTIR of biodegradable sheets.

**Figure 5:**
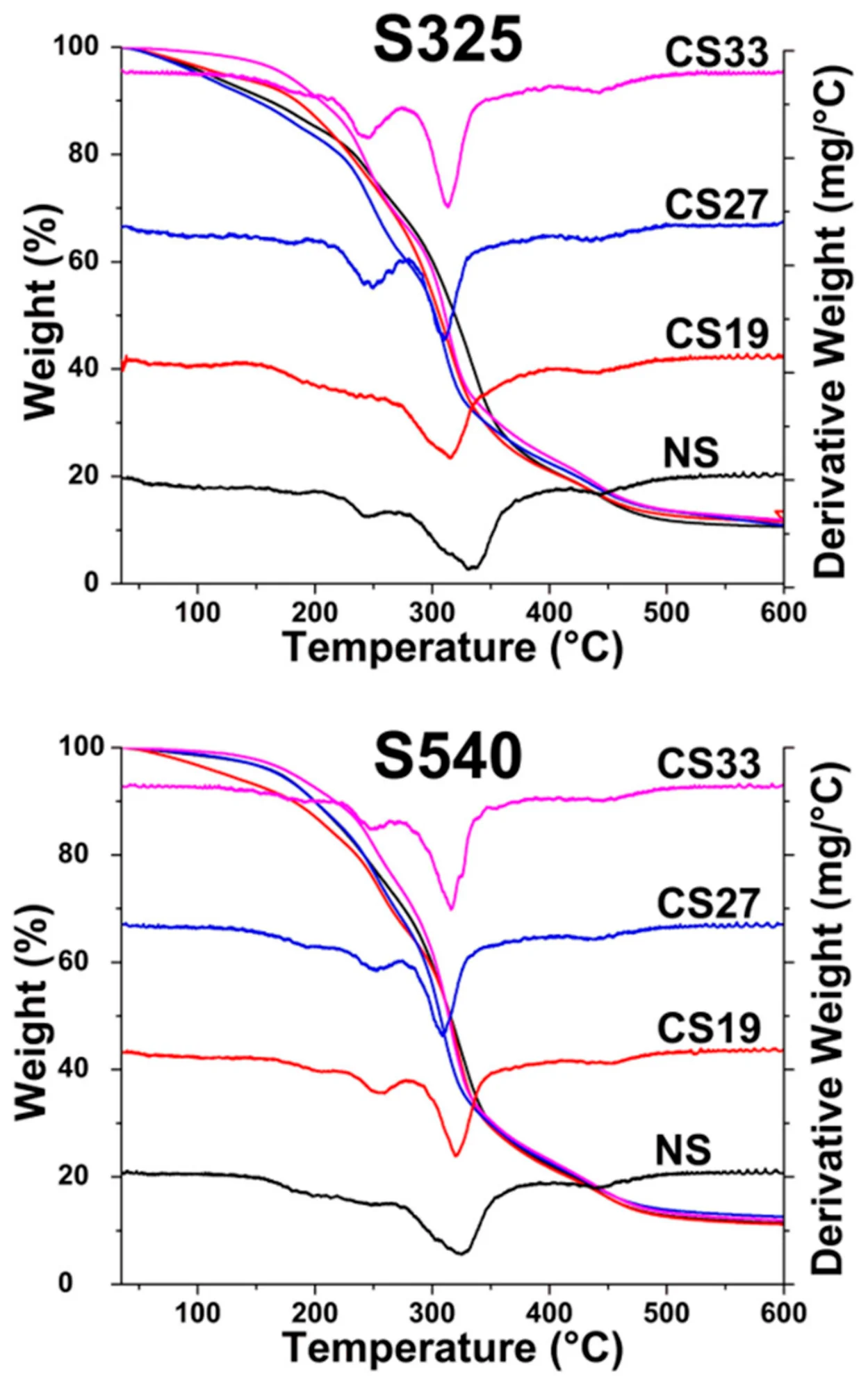
Thermogravimetric analysis of biodegradable sheets.

**Table 1:** Hyperparameters to be optimized.

Model	Hyperparameters
MLP	Number of neurons in the ith hidden layer: (50), (100), (50, 50), and (100, 100); Activation function for the hidden layer: identity, logistic, tanh, and relu; Solver for weight optimization: lbfgs, sgd, and adam; and Learning rate schedule for weight updates: constant, invscaling, and adaptive
DT	Strategy used to choose the split at each node: best and random; Maximum depth of the tree: none, 10, 20, 30, 40, and 50; Minimum number of samples required to split an internal node: 2, 5, 10, 15, and 20; Number of features to consider when looking for the best split; none, auto, sqrt, and log2; Maximum number of leaf nodes: none, 10, 20, 30, 40, and 50
RF	Number of trees in the forest: 10, 50, 100, and 200; Maximum depth of the tree: none, 10, 20, 30, 40, and 50; Minimum number of samples required to split an internal node: 2, 5, 10, 15, and 20; Minimum number of samples required to be at a leaf node 1, 2, 5, 10, and 15; and Maximum number of leaf nodes allowed in each tree of the forest: none, 10, 20, 30, 40
GB	Number of boosting stages to perform: 10, 50, 100, and 200; Learning rate: 0.001, 0.01, 0.1, and 0.2; Minimum number of samples required to split an internal node: 2, 5, 10, 15, and 20; and Maximum depth of the individual regression estimators: 3, 10, 20, 30, 40, and 50
XGB	Number of gradient boosted trees: 10, 50, 100, and 200; Learning rate: 0.001, 0.01, 0.1, and 0.2; Maximum tree depth for base learners: 3, 6, 9, and 12; Minimum sum of instance weight(hessian) needed in a child: 1, 3, 5, and 7; Minimum loss reduction required to make a further partition on a leaf node of the tree: 0.0, 0.1, 0.2, 0.3, and 0.4; Subsample ratio of the training instance: 0.5, 0.75, and 1.0; Subsample ratio of columns when constructing each tree.: 0.5, 0.75, and 1.0; Balancing of positive and negative weights: 1, 2, 3, and 4; Booster: gbtree, gblinear and dart; Tree method: auto, exact, approx, hist, and gpu_hist; Maximum number of leaves: 0, 10, 20, 30, 40, and 50; and Sampling method: uniform and gradient_based
KNN	Number of neighbors: 3, 5, 7, 9, and 11; Weight function used in prediction: uniform and distance; Metric to use for distance computation: Euclidean, Manhattan, and Minkowski

**Table 2:** Water vapor permeability, apparent opacity, color difference, and crystallinity index of the biodegradable sheets.

Starch	PVA	Thickness (μm)	WVP ^1^ (g · m^−1^ · s^−1^ · kPa^−1^) (×10^10^)	Weight Loss in Water (%)	Apparent Opacity (%)	Color Difference (∆E*)	Crystallinity Index (%)
NS	S540	317 ± 64 ^c^	2.8 ± 0.1 ^ab^	37 ± 1 ^bc^	24 ± 2 ^cd^	18 ± 2 ^b^	25.6
	S325	674 ± 112 ^a^	3.2 ± 0.1 ^abc^	42 ± 1 ^abc^	31 ± 1 ^b^	23 ± 1 ^ab^	25.3
CS19	S540	652 ± 76 ^a^	3.4 ± 0.1 ^cd^	44 ± 3 ^abc^	29 ± 1 ^bc^	20 ± 1 ^b^	22.5
	S325	563 ± 135 ^ab^	3.6 ± 0.1 ^cd^	47 ± 2 ^ab^	38 ± 1 ^a^	28 ± 2 ^a^	24.8
CS27	S540	538 ± 88 ^ab^	3.3 ± 0.1 ^bcd^	41 ± 3 ^abc^	22 ± 1 ^d^	12 ± 1 ^c^	24.6
	S325	435 ± 94 ^bc^	3.8 ± 0.2 ^d^	52 ± 1 ^a^	27 ± 1 ^bcd^	20 ± 1 ^b^	26.5
CS33	S540	528 ± 121 ^ab^	3.3 ± 0.1 ^bcd^	34 ± 3 ^c^	28 ± 1 ^bc^	22 ± 1 ^b^	21.9
	S325	673 ± 138 ^a^	2.7 ± 0.1 ^a^	48 ± 1 ^ab^	27 ± 2 ^bcd^	18 ± 1 ^bc^	25.9

^1^ Water vapor permeability. ^a,b,c,d^ means with the same letter at the same column are not different statistically (*p* < 0.05).

**Table 3:** Obtained metrics in tensile strength prediction.

Metric	MLP	DT	RF	GB	XGB	KNN
$R^{2}$	0.865	0.940	0.960	0.976	0.865	0.867
MSE	0.742	0.328	0.220	0.135	0.741	0.730
MAPE (%)	17.04	11.34	7.62	7.130	17.98	11.98
MAE	0.580	0.461	0.325	0.292	0.632	0.602
RMSE	0.861	0.573	0.469	0.367	0.861	0.854

**Table 4:** Obtained metrics in elongation prediction.

Metric	MLP	DT	RF	GB	XGB	KNN
$R^{2}$	0.699	0.917	0.967	0.982	0.976	0.821
MSE	3515.297	971.194	384.382	207.064	276.888	2089.572
MAPE (%)	16.540	7.337	4.931	2.813	3.221	7.161
MAE	55.245	23.775	16.159	10.024	11.582	28.678
RMSE	59.300	31.164	19.606	14.390	16.640	45.712

**Table 5:** Obtained metrics in Young’s modulus prediction.

Metric	MLP	DT	RF	GB	XGB	KNN
$R^{2}$	−0.604	0.963	0.897	0.871	0.574	0.433
MSE	40.337	0.934	2.592	3.237	10.709	14.255
MAPE (%)	49.668	8.076	28.011	35.864	60.898	69.769
MAE	5.215	0.713	1.154	1.689	2.963	2.603
RMSE	6.351	0.966	1.610	1.799	3.272	3.776

**Table 6:** Obtained metrics in Op prediction.

Metric	MLP	DT	RF	GB	XGB	KNN
$R^{2}$	−1.493	0.249	0.497	0.599	0.904	0.744
MSE	51.530	15.527	10.399	8.288	1.982	5.294
MAPE (%)	19.552	10.678	9.509	8.133	4.664	5.760
MAE	6.178	3.153	2.741	2.382	1.356	1.662
RMSE	7.178	3.940	3.225	2.879	1.408	2.31

**Table 7:** Obtained metrics in WVP prediction.

Metric	MLP	DT	RF	GB	XGB	KNN
$R^{2}$	−0.039	−0.240	0.732	0.221	0.061	0.054
MSE	0.099	0.119	0.026	0.074	0.090	0.090
MAPE (%)	8.310	9.834	4.514	8.089	8.388	8.776
MAE	0.264	0.317	0.145	0.259	0.270	0.284
RMSE	0.315	0.344	0.160	0.273	0.300	0.301

**Table 8:** Obtained metrics in WLW prediction.

Metric	MLP	DT	RF	GB	XGB	KNN
$R^{2}$	−1.241	−0.964	−0.626	−0.282	−0.421	0.344
MSE	42.016	36.807	30.485	24.032	26.631	12.291
MAPE (%)	11.698	11.483	9.690	9.052	9.381	6.164
MAE	5.211	5.069	4.332	4.019	4.212	2.810
RMSE	6.482	6.067	5.521	4.902	5.161	3.506

**Table 9:** Obtained metrics in $∆E^{*}$ prediction.

Metric	MLP	DT	RF	GB	XGB	KNN
$R^{2}$	−0.447	0.256	0.316	0.103	0.031	0.315
MSE	27.872	14.337	13.163	17.273	18.651	13.197
MAPE (%)	16.687	15.657	13.385	12.218	12.319	14.421
MAE	3.878	3.324	2.999	2.941	2.924	3.114
RMSE	5.279	3.787	3.628	4.156	4.319	3.633

**Table 10:** Best models and hyperparameters.

Property	Best Model and Hyperparameters
Tensile strength	GB model (Learning rate: 0.2; Maximum depth of the individual regression estimators: 3; Minimum number of samples required to split an internal node: 5; and Number of boosting stages to perform: 200)
Elongation at break	GB model (Learning rate: 0.1; Maximum depth of the individual regression estimators: 3; Minimum number of samples required to split an internal node: 5; and Number of boosting stages to perform: 200)
Young’s modulus	DT model (Maximum depth of the tree: none; Number of features to consider when looking for the best split: none; Maximum number of leaf nodes: 30; Minimum number of samples required to split an internal node: 5; and Strategy used to choose the split at each node: random)
WVP	Did not provide an adequate fit
WLW	Did not provide an adequate fit
Op	XGB model (Tree method: exact; Subsample ratio of the training instance: 0.5; Balancing of positive and negative weights: 3; Sampling method: uniform; Number of gradient boosted trees: 100; Minimum sum of instance weight (hessian) needed in a child: 1; Maximum number of leaves: 30; max_depth: 9; Learning rate: 0.2; Minimum loss reduction required to make a further partition on a leaf node of the tree: 0.3; Subsample ratio of columns when constructing each tree: 0.75; and Booster: dart)
$∆E^{*}$	Did not provide an adequate fit

**Table 11:** Feature importance in properties prediction.

Property	HD	DS
Tensile strength	0.865	0.135
Elongation at break	0.850	0.150
Young’s modulus	0.660	0.340
WVP	0.532	0.468
WLW	0.800	0.200
Op	0.532	0.468
∆E*	0.582	0.418

## Data Availability

The original contributions presented in this study are included in the article/Supplementary Materials. Further inquiries can be directed to the corresponding author.

## Supplementary Materials

The following supporting information can be downloaded at: https://www.mdpi.com/article/10.3390/foods15193360/s1, Table S1. Mechanical Properties of the biodegradable sheets.
