# Supplementary material for: Cationic Starch and Polyvinyl Alcohol Composite Sheets Produced by Extrusion-Calendering
Source: Foods. 2026 Sep 22;15(19):3360. doi: 10.3390/foods15193360 (PMC13664108; doi:10.3390/foods15193360)
Supplement: Supplementary file 1 [file foods-15-03360-s001.zip › foods-4543027-supplementary.pdf]

Supplementary Material

Table S1. Mechanical Properties of the biodegradable sheets

Starch	PVA	Tensile strength (MPa)	Elongation at break (%)	Young's modulus (MPa)
NS	S540	2.0 ± 0.3^{d}	180.2 ± 20.5^{c}	3.4 ± 0.3^{d}
	S325	6.1 ± 0.4^{a}	415.9 ± 25.9^{ab}	13.9 ± 1.1^{a}
CS19	S540	1.2 ± 0.1^{e}	205.0 ± 14.3^{c}	2.7 ± 0.2^{d}
	S325	5.8 ± 0.6^{a}	393.9 ± 27.7^{a}	14.1 ± 0.9^{a}
CS27	S540	0.8 ± 0.1^{e}	132.8 ± 12.6^{c}	2.4 ± 0.3^{e}
	S325	3.9 ± 0.4^{c}	286.6 ± 20.3^{b}	13.0 ± 1.2^{c}
CS33	S540	1.0 ± 0.1^{e}	138.4 ± 14.0^{c}	2.9 ± 0.2^{e}
	S325	4.8 ± 0.5^{b}	349.8 ± 35.4^{ab}	13.6 ± 0.9^{b}
